# Photobiomodulation Therapy in the Treatment of Chronic Dysphagia Post Hormonal Therapy in a Breast Cancer Patient

**DOI:** 10.3390/dj7020053

**Published:** 2019-05-13

**Authors:** Marwan El Mobadder, Fadi Farhat, Samir Nammour

**Affiliations:** 1 Department of Dental Science, Faculty of medicine, University of Liège, 4000 Liège, Belgium; S.Namour@ulg.ac.be; 2 Department of Hematology–Oncology, Hammoud Hospital University Medical Centre, G. Hammoud Street, Sidon 652, Lebanon; drfadi.research@gmail.com

**Keywords:** photobiomodulation, dysphagia, cancer, breast cancer, supportive care

## Abstract

Among the few supportive care measures available for the management of dysphagia, Photobiomodulation (PBM) therapy, defined as the therapeutic use of light, has shown significant promise. In this case report, effective management of chronic dysphagia post hormonal therapy in a breast cancer patient was made. Experts in the supportive care in cancer and PBM proposed and requested further investigations of the protocol used in this case report in the management of dysphagia. In this case report, the protocol of PBM was proposed by experts in supportive care in cancer. Functional outcome swallowing scale for staging oropharyngeal dysphagia was used to assess the effectiveness of the treatment in pre-operative, per and post-operative stage. This case reports that PBM is effective in the management of dysphagia, a side effect of hormonal therapy in a cancer patient.

## 1. Introduction

Swallowing is considered a complex biomechanical interaction of anatomy and physiology in which the precise coordination of over twenty-five pairs of muscles in the oral cavity, pharynx, larynx, and esophagus is required [1]. This complex biomechanical interaction consists of four stages: oral preparatory, oral, pharyngeal, and esophageal. Dysphagia is an impairment of swallowing that may involve any structure from the lips to the gastric cardia [2]. In a cancer patient, dysphagia is considered a debilitating and potentially life-threatening complication that can seriously affect the quality of life (QoL) of patients and can negatively affect their adherence to cancer therapy [3]. Dysphagia is seen as a result of malignancy in the head and neck region, as a side effect of head and neck cancer therapy and as a complication of any cancer therapy modality. In fact, dysphagia can be seen in 50.9% of patients with pharyngeal cancer, in 20% of high-dose radiotherapy patients, in up to 40% of patients treated with concurrent chemotherapy for lung cancer, and according to Sonis et al, 8.5% of patients who developed mammalian target of rapamycin-associated stomatitis [4]. This complication highly affects the quality of life of patients, increases the use of health care resources and costs, and may compromise patient adherence to cancer therapy protocols. Pathogenesis of dysphagia is complex and is associated with acute inflammation, edema, and fibrosis with the injury of the muscle. In addition, dysphagia can be a result of generalized weakness, lack of muscle coordination while swallowing, and excessive fibrosis which will result in loss of elasticity [4,5]. 

Among the few supportive care measures available for the management of dysphagia, Photobiomodulation (PBM), that is the therapeutic use of light, has shown significant promise [6]. Visible, infrared and near infrared light is absorbed by the endogenous chromophores which will trigger biological reactions leading to a physiological change. The mechanism of action of PBM is fundamentally considered as depending on light-mitochondria interactions, likely through a stimulation of mitochondrial respiration and consequent production of reactive oxygen species (ROS). There is a tight relationship between ROS release, mainly by mitochondria, and Ca^2+^ signaling, with ROS regulating Ca^2+^ signaling and Ca^2+^ signaling affecting mitochondrial activities and ROS production [7]. However, it is now established that the primordial mechanism of action of PBM is predominantly related to an action on cytochrome c oxidase (CcO) in the mitochondrial respiratory chain by facilitating electron transport [8]. Additionally, PBM was shown to stimulate collagen production, promote DNA and protein synthesis, modulate cell migration and proliferation [7]. PBM improves wound repair and tissue regeneration by acting on different phases of injury resolution, including inflammation, proliferation, and remodeling phases [9]. Currently, a large number of studies have suggested the effectiveness of PBM in the treatment of oral mucositis as a side effect of cancer treatment [10,11,12] and may have a potential role in the management of acute and chronic dysphagia [13]. Furthermore, an international multidisciplinary panel of clinicians and researchers, with expertise in supportive care in cancer and photobiomodulation, proposed a preventive and curative protocol of PBM for the management of dysphagia. They concluded that further investigations must be done in order to assess the effectiveness of the suggested protocol [6,8,9]. Therefore, the aim of this case report was to note the reduction of chronic dysphagia caused by cancer therapy within this protocol. 

## 2. Case Report 

A 45-year-old female patient with a history of breast cancer, previously treated by left mastectomy 4.5 years ago, chemotherapy 2.5 years ago and radiotherapy 1.5 years ago, started third -line hormonal treatment with Everolimus (AFINITOR 10 mg) and Exemestane (AROMASINE 25 mg) in addition to Zoledronic acid (ZOMETA 4 mg/100 ml). After three months of hormonal therapy, the patient complained of chronic impairment in swallowing in the upper gastrointestinal tract, from the lips to the upper esophageal sphincter. The patient was diagnosed with chronic dysphagia by a certified speech-language therapist. For the assessment of the severity of dysphagia and the follow-up, a functional outcome swallowing scale (FOSS) for staging oropharyngeal dysphagia was used [14]. The treatment of choice was the therapeutic use of photobiomodulation. One session of PBM was given every 24 hours for five days. The FOSS scale was used to measure the severity of dysphagia before the treatment, per-treatment and after 15 days of treatment. Diagnosis revealed that the patient had compensated abnormal function manifested by significant dietary modifications and prolonged mealtime with a stable weight, occasional cough, with an absent aspiration. No ethical committee approval was necessary for our research since the protocol used in this case report is described in literature. The patient signed a written informed consent before enrolling in the study. 

**i Assessment of Dysphagia**

The functional outcome swallowing scale for staging oropharyngeal dysphagia, proposed by John. R Salassa in 1999, was used for the assessment of dysphagia before treatment (T0), at day two of treatment (T2), immediately after treatment (T5) and fifteen days after treatment (T15). The FOSS was at stage II before the treatment (Table 1).

**ii Treatment Protocol of Dysphagia**

The photobiomodulation therapy protocol and parameters used was based on evidence derived from the literature and expert opinion that provided a guideline for the use of photobiomodulation in supportive cancer care for dysphagia (Table 2). Intraoral and extraoral application of diode laser 980 nm (FONA Laser Sirona Dental Systems GmbH, Germany) was used. Intraoral application of the diode laser 980 nm was made with an energy density of 3 J/cm^2^ for 10 seconds at each point, in a continuous and non-contact mode, tip of 320 µm. Bilaterally four points on the soft palate, bilaterally four points on the oropharynx. Extra-oral application was made with an energy density of 3 J/cm^2^, 10 seconds of irradiation on each point, tip of 320 µm, in a continuous and non-contact mode. The irradiation was made on the lateral and ventral pharynx and larynx, midline neck and lateral neck anterior to sternocleidomastoid muscle (Table 2).

**iii Results**

At T0, the FOSS was at stage II. After three sessions of photobiomodulation therapy (T3), there was occasional cough, symptomatic, a little difficulty in swallowing and prolonged meal time with the sensation of food getting stuck in the throat or esophagus (between stage II and I). After the treatment (T5), the patient had complete physiological function and was asymptomatic. Therefore, at stage 0 of FOSS, which persisted after 15 days (T15). A significant reduction of dysphagia (from stage II to Stage 0) was noted after 5 sessions of PBM with the protocol used. Therefore, PBM therapy successfully treated the hormonal therapy induced dysphagia (Table 3).

## 3. Discussion 

Dysphagia may result in numerous important sequelae such as weight loss, aspiration, pneumonia and chronic bronchial inflammation [1]. Aspiration is defined as the passage of materials below the true vocal cord and is generally manifested by cough or clearing of the throat before, during or after swallowing. A certain degree of aspiration may be tolerated by patients, and in other conditions, silent aspiration can be noted in patients with head and neck cancer [2]. Hence, dysphagia has a negative effect on the quality of life along with life-threatening pulmonary complications [6]. Dysphagia is a relatively frequent oral complication of cancer therapy that requires preventive or curative management [15]. In this case, it is notable that dysphagia was not associated with oral mucositis or hyposalivation. In fact, it was the only oral complication diagnosed. Swallowing exercises is one of the promising types of management in preserving swallowing function [16]. A plethora of studies reveal the effectiveness of PBM for preventive and curative treatment of oral mucositis, but only a few have studied its effectiveness on dysphagia [6]. In fact, the use of PBM for the management of chronic dysphagia needs to be explored. In this case, the cancer patient suffered from chronic dysphagia as a side effect of hormonal therapy, this finding was rare in literature, which was significant, since dysphagia is a frequent oral complication of head and neck radiotherapy. In this case report, five sessions of PBM therapy were sufficient to completely treat Stage II dysphagia. These findings can be explained by the ability of PBM to prevent and ameliorate inflammation and pain associated with oral mucositis, along with its potential to control exuberant fibrosis. On the other hand, Eisbruch et al. identified that irritations of the tongue base, pharyngeal constrictors, the larynx, and the autonomic neural plexus is crucial in the development of dysphagia which can explain the positive outcome of the study [17]. In this case report, the irradiated zone were the soft palate, pharynx, larynx and oropharynx. A triple blinded randomized controlled trial reported a lower incidence of severe oral mucositis and dysphagia when predetermined oral sites were exposed to PBM prior to and during radiotherapy [7]. Within the limitations of this case report, PBM was able to treat dysphagia induced by hormonal therapy in a breast cancer patient. Further investigations must be made.

## 4. Conclusions

It can be concluded that the therapeutic use of photobiomodulation, according to specific protocol, is considered a good approach for the treatment of dysphagia induced by hormonal therapy in a breast cancer patient.

## Figures and Tables

**Table 1:** Functional outcome swallowing scale for staging oropharyngeal dysphagia proposed by John R. Salassa in the 39th annual meeting of the American society for head and neck surgery (14).

Stage	Stage Criteria
Stage 0	Normal physiological function and asymptomatic
Stage I	Normal physiological function but with episodic or daily symptoms of dysphagia such as reflux symptoms, globus, odynophagia, repetitive swallow, throat-clearing habit, difficulty chewing, minor oral incompetence, sensation of food getting stuck in the throat or esophagus.
Stage II	Compensated abnormal function manifested by significant dietary modifications or prolonged mealtime. Weight is stable, cough is absent or occasional, aspiration is absent or occasional and mild.
Stage III	Decompensated abnormal function manifested by weight loss of 10% or less of body weight over 6 months due to dysphagia, or frequent cough, gagging, or aspiration during meals. Aspiration may be mild or moderate. Patients in this stage are unstable in terms of nutrition or respiratory status. Pulmonary complications have not occurred, but the patient is at risk.
Stage IV	Severely decompensated abnormal function manifested by weight loss of more than 10% of body weight over 6 months due to dysphagia, or severe aspiration. Non-oral feeding recommended for most (>50%) of nutrition. Patients in this stage are nearly complete failures at swallowing and may safely swallow only under strictly defined conditions which do not meet their nutritional needs
Stage V	Nonoral feeding for all nutrition. Patients in this stage are complete failures at swallowing. They are different from stage IV in that they cannot swallow anything safely.

**Table 2:** Photobiomodulation protocol, parameters and zone irradiated for the treatment of dysphagia (6).

Irradiation	Treatment Area	Parameters
Intraoral	Four points on the soft palate, four points on the oropharynx. Bilaterally, four points to soft palate and onto oropharynx	Wavelength of 980 nm, 3 J/cm^2^ for 10 seconds on each point, tip of 320 µm, continuous and contact mode.
Extraoral	Lateral and ventral pharynx and larynx. Midline neck and lateral neck anterior to sternocleidomastoid muscle	Wavelength of 980 nm, 3 J/cm^2^ for 10 seconds on each point, tip of 320 µm, continuous and contact mode.

**Table 3:** Results of the assessments of dysphagia according to the functional outcome swallowing scale for staging oropharyngeal dysphagia.

Complication	T initial	T3	T5	T15
Dysphagia stage according to FOSS	Stage II	Between II and I	Stage 0	Stage 0

FOSS = Functional outcome swallowing scale for staging oropharyngeal dysphagia, T initial = before treatment, T3 = three days after treatment, T5 = five days after treatment, T15 = fifteen days after treatment.

## References

[1] Leslie P., Carding P.N., Wilson J.A. (2003). Investigation and management of chronic dysphagia. BMJ.

[2] Murphy B.A., Gilbert J. (2009). Dysphagia in head and neck cancer patients treated with radiation: Assessment, sequelae, and rehabilitation. Semin. Radiat. Oncol..

[3] Cooperstein E., Gilbert J., Epstein J.B., Dietrich M.S., Bond S.M., Ridner S.H., Wells N., Cmelak A., Murphy B.A. (2012). Vanderbilt Head and Neck Symptom Survey version 2.0: Report of the development and initial testing of a sub scale for assessment of oral health. Head Neck.

[4] Russi E.G., Corvò R., Merlotti A., Alterio D., Franco P., Pergolizzi S., De Sanctis V., Redda M.G., Ricardi U., Paiar F. (2015). Swallowing dysfunction in head and neck cancer patients treated by radiotherapy: Review and recommendations of the supportive task group of the Italian Association of Radiation Oncology. Cancer Treat. Rev..

[5] Cullins M.J., Connor N.P. (2019). Reduced tongue force and functional swallowing changes in a rat model of post stroke dysphagia. Brain Res..

[6] Zecha J.A., Raber-Durlacher J.E., Nair R.G., Epstein J.B., Elad S., Hamblin M.R., Barasch A., Migliorati C.A., Milstein D.M., Genot M.T. (2016). Low-level laser therapy/photobiomodulation in the management of side effects of chemoradiation therapy in head and neck cancer: Part 2: Proposed applications and treatment protocols. Support. Care Cancer.

[7] Amaroli A., Ravera S., Baldini F., Benedicenti S., Panfoli I., Vergani L. (2018). Photobiomodulation with 808-nm diode laser light promotes wound healing of human endothelial cells through increased reactive oxygen species production stimulating mitochondrial oxidative phosphorylation. Lasers Med. Sci..

[8] El Mobadder M., Farhat F., El Mobadder W., Nammour S. (2018). Photobiomodulation Therapy in the Treatment of Oral Mucositis, Dysgeusia and Oral Dryness as Side-Effects of Head and Neck Radiotherapy in a Cancer Patient: A Case Report. Dent. J..

[9] Zecha J.A., Raber-Durlacher J.E., Nair R.G., Epstein J.B., Sonis S.T., Elad S., Hamblin M.R., Barasch A., Migliorati C.A., Milstein D.M. (2016). Low level laser therapy/photobiomodulation in the management of side effects of chemoradiation therapy in head and neck cancer: Part 1: Mechanisms of action, dosimetric, and safety considerations. Support. Care Cancer.

[10] Oberoi S., Zamperlini–Netto G., Beyene J., Treister N.S., Sung L. (2014). Effect of prophylactic low level laser therapy on oral mucositis: A systematic review and meta-analysis. PLoS ONE.

[11] Bensadoun R.J., Nair R.G. (2012). Efficacy of low-level laser therapy (LLLT) in oral mucositis: What have we learned from randomized studies and meta-analyses?. Photomed. Laser Surg..

[12] Fekrazad R., Chiniforush N. (2014). Oral mucositis prevention and management by therapeutic laser in head and neck cancers. J. Lasers Med. Sci..

[13] Elting L.S., Keefe D.M., Sonis S.T., Garden A.S., Spijkervet F.K., Barasch A., Tishler R.B., Canty T.P., Kudrimoti M.K., Vera-Llonch M. (2008). Burden of Illness Head and Neck Writing Committee. Patient-reported measurements of oral mucositis in head and neck cancer patients treated with radiotherapy with or without chemotherapy: Demonstration of increased frequency, severity, resistance to palliation, and impact on quality of life. Cancer.

[14] King S.N., Dunlap N.E., Tennant P.A., Pitts T. (2016). Pathophysiology of radiation-induced dysphagia in head and neck cancer. Dysphagia.

[15] Wells M., King E. (2017). Patient adherence to swallowing exercises in head and neck cancer. Curr. Opin. Otolaryngol. Head Neck Surg..

[16] Eisbruch A., Schwartz M., Rasch C., Vineberg K., Damen E., Van As C.J., Balm A.J. (2004). Dysphagia and aspiration after chemoradiotherapy for head-and-neck cancer: Which anatomic structures are affected and can they be spared by IMRT. Int. J. Radiat. Oncol. Biol. Phys..

[17] Gautam A.P., Fernandes D.J., Vidyasagar M.S., Maiya A.G., Vadhiraja B.M. (2012). Low level laser therapy for concurrent chemoradiotherapy induced oral mucositis in head and neck cancer patients-a triple blinded randomized controlled trial. Radiother. Oncol..

